# Case series: acupuncture-related pneumothorax

**DOI:** 10.1186/s12245-022-00455-z

**Published:** 2022-09-12

**Authors:** Francesca Th’ng, Kailing Adriel Rao, Po Yu Huang

**Affiliations:** Acute and Emergency Care Department, Khoo Teck Puat Hospital, Singapore, Singapore

**Keywords:** Acupuncture, Pneumothorax, Complimentary medicine, Traditional Chinese medicine

## Abstract

**Background:**

Acupuncture has become a more popular complementary and alternative medicine worldwide. As pneumothorax is a rare acupuncture-related adverse event (AE), it is thought to be under-recognized by acupuncturists and emergency physicians, and the public is often not aware of this potential AE resulting in delayed hospital presentation.

**Methods:**

This is a case series of acupuncture-related pneumothoraces diagnosed in an emergency department (ED) in Singapore. Data was collected retrospectively from patients’ notes and prospectively from the patients over the phone.

**Case presentations:**

Between 2017 and 2021, 4 out of 474 (0.84%) pneumothoraces were acupuncture related. Three of these patients consented to participate in this study. One patient developed bilateral pneumothoraces. All 3 patients claimed that they were not informed by the acupuncturists of potential serious AEs prior to acupuncture treatments and that they were not aware that such AE could occur. All 3 patients had reported their symptoms of chest pain and/or breathlessness to their acupuncturists post-treatment, but they were not advised to seek urgent medical attention. When the 3 patients had informed their acupuncturists about their diagnosis of pneumothorax, 2 of the acupuncturists did not seem to be aware of this acupuncture-related AE.

**Discussion:**

When pneumothorax manifests, there is a potential need for an invasive procedure and continuous monitoring as it may devolve into a life-threatening condition with cardiovascular compromise. Early medical recognition and attention are needed to ensure optimal patient outcomes. In the appropriate population cohort, a history of prior acupuncture treatments should be included as part of history-taking assessment in patients presenting with chest pain and/or breathlessness.

**Conclusion:**

Emergency physicians should be vigilant of this potentially serious and life-threatening complication for anyone presenting with chest discomfort and/or breathlessness after recently undergoing acupuncture to ensure earlier diagnosis, management, and better patient outcome.

## Background

Acupuncture has been practiced as part of traditional Chinese medicine for centuries. Over the past decade or so, acupuncture has become a more popular complementary and alternative medicine worldwide. However, there are still uncertainties about the safety of acupuncture. The incidence of acupuncture-related adverse events (AEs) has been reported to be 3.76–8.6%. The common potential AEs include subcutaneous hematoma, minor hemorrhage in the needling position, subcutaneous bruise, prolonged pain at the site of needling, fainting, abdominal distension, dizziness/vertigo, leg weakness, and muscle spasm [1, 2].

Serious and potentially life-threatening AEs related to acupuncture are rarely reported, but this includes the transmission of infections, pneumothorax, cardiovascular lesions, and hemorrhage or hematomas of the central nervous system [3]. The real incidence of acupuncture-related pneumothorax is not known. A systematic review by Ernst et al. [4] reported that pneumothorax was the most frequent serious AE accounting for 4 deaths amongst 95 patients. Conversely, a prospective observational study in Germany [2] demonstrated that pneumothorax occurred twice in nearly a quarter of a million treatments.

We present a case series of 3 acupuncture-related pneumothoraces seen in our Emergency Department (ED) at Khoo Teck Puat Hospital (KTPH), Singapore. With this case series, we hope that pneumothoraces post acupuncture could be diagnosed and ultimately managed more efficiently by emergency physicians. In addition, we hope to raise public awareness so that patients would seek medical care sooner for their symptoms to ensure better outcomes.

## Methods

KTPH is a 795-bed acute hospital in the North of Singapore and serves more than 550,000 people in the region. Between the 1st of January 2017 and the 31st of December 2021, the ED treated 474 patients with a diagnosis of pneumothorax of all etiology. Four patients (0.84%) had a documented history of receiving acupuncture treatment prior to their symptoms of chest discomfort and/or breathlessness. Three of these patients consented to participate in this case series. Verbal consent was obtained from patients over the phone. Patient information was collected retrospectively from patients’ notes and prospectively over the phone.

## Case presentations

### Case 1

Mr. A, a 40-year-old non-smoker, had been undergoing acupuncture treatments over the past year for his muscle aches. Acupuncture needles were normally inserted over his bilateral chest walls between the ribs, and there were no complications during prior treatments. On the acupuncture day prior to the ED visit, he felt a sudden sharp pain upon needle insertion over his left chest wall and had screamed aloud. The acupuncturist subsequently retracted the needle slightly and proceeded to complete the treatment. Shortly after, Mr. A developed a “hollow” sensation within his chest and left-sided chest pain. On day 3 of his symptoms, he had informed his acupuncturist of his symptoms but was advised to rest. Mr. A decided to go to the ED on day 3 as his chest pains were getting worse. At ED, his vital signs were stable (pulse: 87 beats per minute, blood pressure (BP): 132/93 mmHg, respiratory rate (RR): 18 beats per minute, SpO_2_: 100% on room air, and temperature (T): 36.8°C). His chest X-ray showed a left-sided pneumothorax with a 1-cm apicopleural distance. He was managed conservatively with oxygen therapy and was subsequently discharged the next day.

### Case 2

**Fig. 1:**
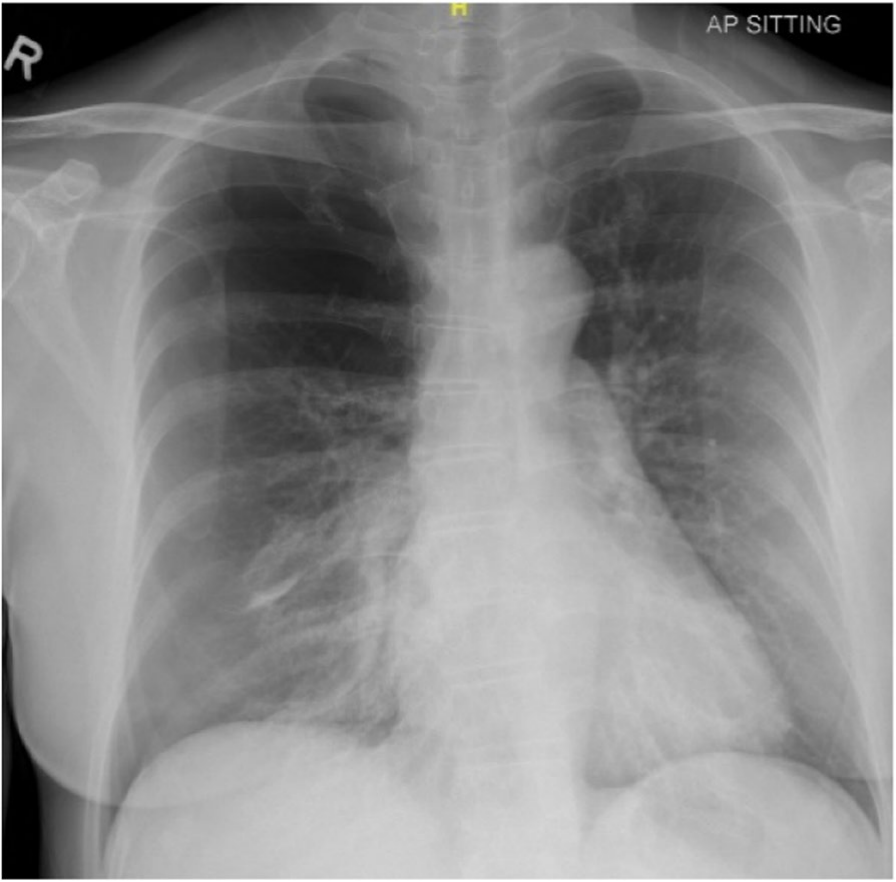
Chest X-ray showing right-sided pneumothorax with apicopleural distance of 7.8cm and left apical pneumothorax with apicopleural distance of 1.7cm. There is no tracheal deviation or mediastinum shift

**Fig. 2:**
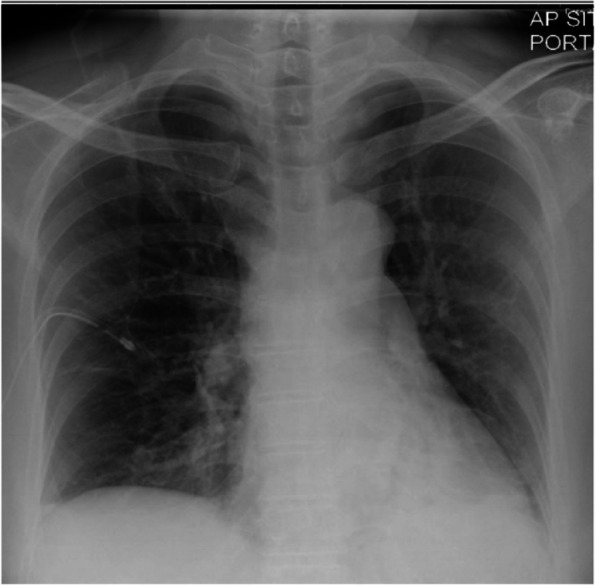
Chest X-ray after insertion of Seldinger chest drain showing reduced small right pneumothorax with a width of about 1.5cm at the apex. A small left apical pneumothorax is still present

Mrs. B, a 50-year-old non-smoker, had been undergoing acupuncture over the past 3 months for her benign paroxysmal positional vertigo (BPPV). She claimed that the acupuncture needles would normally be inserted throughout her “whole body” including her back and bilateral chest wall. After her treatment in the morning, she had developed severe right-sided chest pain and breathlessness by that night. She informed her acupuncturist the next day and was advised to rest. By that evening of day 2, her symptoms had gotten so severe that she could barely walk and was brought to the ED by her daughter. In the ED, her vital signs were normal (pulse: 84, BP 128/75, RR: 18, SpO_2_: 100% on air, T: 37.2°). Her chest X-ray showed a right-sided pneumothorax with a 7.8-cm apicopleural distance and a left-sided pneumothorax with a 1.7-cm apicopleural distance (Fig. 1). A right-sided Seldinger chest drain was inserted, and she was admitted to the respiratory ward (Fig. 2). On the ward, Mrs. B’s pneumothoraces improved in size and she was discharged 3 days later.

### Case 3

Mrs. C, a 70-year-old non-smoker with a background of diabetes and hypertension, had been receiving acupuncture treatment for years to relieve her shoulder muscle aches. She claimed that the acupuncture needles were normally inserted over her bilateral trapezius and shoulder regions. On that day, she felt that the needles over her left trapezius were inserted deeper than usual. She felt some bearable pain over her left chest wall which radiated to her back during the treatment. About 2 h later, she developed breathlessness. She went back to the acupuncturist that day and was told to “relax.” Mrs. C decided to attend the ED on day 1 of her symptoms. At the ED, her vital signs were stable (pulse: 87, BP: 155/95, RR: 18, SpO_2_: 96% on room air, T: 36.6°C). Her chest X-ray showed a left-sided pneumothorax with apicopleural distance of 3cm. She was managed conservatively and was admitted to the respiratory ward. Her pneumothorax reduced in size with oxygen therapy, and she was eventually discharged on day 4.

In this case series, all 3 patients claimed that they were not informed by the acupuncturists of potential serious AEs prior to their acupuncture treatments and that they were not aware that such AEs including pneumothorax could occur. All 3 patients had reported their symptoms to their acupuncturists, but the patients were not advised to seek medical attention. When the 3 patients subsequently informed their acupuncturists about their diagnosis of pneumothorax, 2 of the acupuncturists did not seem to be aware of this AE.

## Discussion

We think that pneumothorax is under-recognized by acupuncturists, emergency physicians, and the public as an acupuncture-related AE. In our population cohort, acupuncture-related pneumothorax is not as rare as previously thought. Furthermore, the incidence of such AEs may actually be higher as emergency physicians may not have asked specifically about the history of prior acupuncture treatments due to the lack of awareness.

When pneumothorax manifests, there is a potential need for an invasive procedure and continuous monitoring as it may devolve into a life-threatening condition with cardiovascular compromise. Early medical recognition and attention are needed to ensure the optimal patient outcome. In the appropriate population cohort, a history of prior acupuncture treatments should be included as part of the history-taking assessment in patients presenting with chest pain and/or breathlessness. Factors that have been recognized to be associated with an increased risk of developing pneumothorax post acupuncture treatment include smoking, being a tall male, having emphysema, consuming corticosteroids, and having active cancer [5]. Interestingly, none of the 3 patients in this case series had any of those factors. We therefore recommend that education of such AEs related to acupuncture be delivered to acupuncturists, emergency physicians, and the public.

Two of the patients in this case series had delayed presentations to the ED. Mr. A presented to ED on day 3 and Mrs. B on day 2. We speculate that if Mrs. B had presented to the ED on day 1 of her symptoms, her pneumothoraces may have been smaller in size upon diagnosis and she could have been managed conservatively. The current Advanced Trauma Life Support (ATLS) [6] guidance recommends the insertion of a chest drain to manage a traumatic pneumothorax. However, there is currently no specific guidance on the management of smaller traumatic pneumothoraces in hemodynamically stable patients. An observational study [7] amongst 602 patients demonstrated that >90% of patients whose traumatic pneumothorax was managed conservatively did not require subsequent chest tube insertion. The median pneumothorax size that was successfully managed conservatively in the study was 5.3 mm (IQR ± 8.6) and that had failed conservative management was 8.2 mm (IQR ±16.5). In comparison, a study carried out by Figueroa et. al. [8] demonstrated that traumatic pneumothoraces ≤ 35 mm, without hemothorax, could be safely treated conservatively in hemodynamically normal patients. Such an initial conservative approach may reduce unnecessary chest drain insertions which too come with its own complications and side effects.

The Traditional Chinese Medicine Practitioners Board (TCMPB) [9] is a statutory board established under the traditional Chinese medicine (TCM) practitioners act. In Singapore, TCM practitioners including acupuncturists are required to be licensed by the TCMPB since 2000 before they are allowed to practice. The TCMPB also regulates the professional ethics and conduct of registered TCM practitioners and has suspended and cancelled registrations of errant TCM physicians. Since April 2020, compulsory Continuing Professional Education (CPE) has also been implemented for all fully and conditionally registered TCM practitioners to meet CPE requirements before their practicing certificates can be renewed. There is currently no statutory requirement for acupuncturists to report AEs, although public healthcare institutions and members of the public are able to report AEs or lodge complaints to TCMPB regarding their acupuncture treatment(s) performed.

## Conclusion

Emergency physicians should be vigilant of this potentially serious and life-threatening complication for anyone presenting with chest discomfort and/or breathlessness after recently undergoing acupuncture to ensure earlier diagnosis, management, and better patient outcome.

## Data Availability

The data collected during the current study are available from the corresponding author upon reasonable request.
